# Prevalence and Spectrum of Predisposition Genes With Germline Mutations Among Chinese Patients With Bowel Cancer

**DOI:** 10.3389/fgene.2021.755629

**Published:** 2022-01-27

**Authors:** Zhengyong Xie, Yongli Ke, Junyong Chen, Zehang Li, Changzheng Wang, Yuhong Chen, Hongliang Ding, Liyang Cheng

**Affiliations:** General Surgery Department, General Hospital of Southern Theatre Command, People’s Liberation Army of China (PLA), Guangzhou, China

**Keywords:** bowel cancer, germline, somatic, P/LP (pathogenic/likely-pathogenic), TMB, DDR

## Abstract

**Background:** Bowel cancer is the third-most common cancer and the second leading cause of cancer-related death worldwide. Bowel cancer has a substantial hereditary component; however, additional hereditary risk factors involved in bowel cancer pathogenesis have not been systematically defined.

**Materials and Methods:** A total of 573 patients with bowel cancer were enrolled in the present study, of whom 93.72% had colorectal cancer (CRC). Germline mutations were integrated with somatic mutation information via utilizing target next-generation sequencing.

**Results:** Pathogenic/Likely Pathogenic (P/LP) germline alterations were identified in 47 (8.2%) patients with bowel cancer and the ratio of the number of these patients with family history was significantly higher in the P/LP group than that noted in the non-pathogenic (Non-P) group. Certain rare germline alterations were noted, such as those noted in the following genes: *FANCD2, CDH1*, and *FLCN*. A total of 32 patients (68.1%) had germline alterations in the DNA-damage repair (DDR) genes and homologous recombination (HR) accounted for the highest proportion of this subgroup. By comparing 573 patients with bowel cancer with reference controls (China_MAPs database), significant associations (*p* < 0.01) were observed between the incidence of bowel cancer and the presence of mutations in *APC, ATM, MLH1, FANCD2, MSH3, MSH6, PMS1*, and *RAD51D*. Somatic gene differential analysis revealed a marked difference in 18 genes and a significant difference was also noted in tumor mutation burden (TMB) between germline mutation carriers and non-germline mutation subjects (*p* < 0.001). In addition, TMB in DDR mutation groups indicated a dramatic difference compared with the non-DDR mutation group (*p* < 0.01). However, no statistically significant differences in TMB were noted among detailed DDR pathways for patients with bowel cancer, irrespective of the presence of germline mutations. Moreover, a significantly higher level (*p* < 0.0001) of mutation count was observed in the DDR group from The Cancer Genome Atlas (TCGA) database and the DDR and non-DDR alteration groups displayed various immune profiles.

**Conclusion:** Chinese patients with bowel cancer exhibited a distinct spectrum of germline variants, with distinct molecular characteristics such as TMB and DDR. Furthermore, the information on somatic mutations obtained from TCGA database indicated that a deeper understanding of the interactions among DDR and immune cells would be useful to further investigate the role of DDR in bowel cancer.

## Introduction

Bowel cancer ranks third with regard to cancer morbidity and mortality worldwide (Thanikachalam and Khan, 2019). According to the Chinese Cancer Registration Report of 2018, 387,600 bowel cancer new cases and 187,100 bowel cancer-related deaths occurred in China during 2015, ranking it the fourth (9.87%) and fifth (8.01%) highest incidence and mortality rates, respectively, among all cancers (Arnold et al., 2020; Yang et al., 2020). In addition, the rates of bowel cancer steadily increased from 2000 to 2018 (Bhui et al., 2009; Bray et al., 2018). Although lower rates compared with the world average (incidence rate of 17.81/100,000 persons and mortality rate of 8.12/100,000 persons) (Bray et al., 2018), the number of new bowel cancer cases and bowel cancer-related deaths in China is the highest in the world due to its relatively large population. Genetic factors resulting in the early development of cancers account for a substantial number of bowel cancer (Medina Pabón and Babiker, 2021). Therefore, it is necessary to explore the prevalence of hereditary bowel cancer and the contribution of the pathogenic germline variants in the development of this disease in the Chinese population.

In general, hereditary cancer syndromes have been implicated in 3–5% of overall cases with bowel cancer (Medina Pabón and Babiker, 2021). Individuals who harbor germline mutations in specific genes are at high risk for developing bowel cancer. Clinically, individuals with hereditary bowel cancer syndromes may be alert to this situation and more likely to undertake frequent early screening (Sokic-Milutinovic and eng, 2019). It is known that germline mismatch repair (MMR) gene mutations, together with *APC* gene mutations, contribute significantly to inherited bowel cancer (Sa et al., 2020). Lynch Syndrome, the most common hereditary cancer syndrome associated with predisposition to bowel cancer, is associated with germline mutations in DNA mismatch repair (MMR) genes such as *MLH1, MSH2, MSH6, PMS2,* and *EPCAM* (Engel et al., 2020)*.* Familial adenomatous polyposis (FAP) is associated with germline mutations in the *APC* tumor suppressor gene and has been implicated in 1% of cases with bowel cancer. Germline mutations in additional high and moderate penetrance cancer genes such as *BRCA1, CDH1,* and *MUTYH*, have also been associated with increased risk for the developing colorectal neoplasia (Ma et al., 2018). Recent studies have demonstrated that germline variants in various cancer predisposition genes have been identified in 1 out of 10 adults and children diagnosed with advanced cancer types, as well as those with colorectal (Mueller et al., 2021), pancreatic (Gentiluomo et al., 2020), and metastatic prostate (Giri et al.eng, 2019) cancers. The impact of an individual germline variant for clinical decision-making depends on the specific characteristics of the variant (Bertelsen et al., 2019), which classify whether the variant is pathogenic/likely pathogenic (P/LP), or whether it is known and/or it is likely to affect the function of its gene.

Accurate interpretation of genetic test results is of vital importance, notably for patients who are identified with one or more P/LP germline variant associated with a hereditary cancer syndrome. Due to the development of next-generation sequencing (NGS) technology, it has been found that various ratio of patients with bowel cancer harbor germline mutations (Bien et al., 2019). However, despite germline variants in genes related to cancer susceptibility being more common than initially expected, identification of germline mutations of Chinese patients with bowel cancer and the correlation between germline mutations and somatic mutations has not been studied in detail. The present study sought to determine the characteristics of P/LP germline variants in Chinese patients with bowel cancer. The results revealed that a wider panel of predisposition genes are recommended for Chinese patients with bowel cancer, which will be helpful to aid the establishment of prevention and surveillance strategies that can be used to reduce the incidence of this disease.

## Materials and Methods

### Samples Source and Ethic Data

Patients with bowel cancer gave written informed consent prior to their participation in General Hospital of Southern Theatre Command, PLA. Formalin-fixed, paraffin-embedded (FFPE) tumor tissues and matched blood samples in EDTA tubes (for germline tests) from 573 diagnosed bowel cancer patients (Information on clinicopathological status of patients is provided in Supplementary Table S2) were collected. All tumor FFPE sections were evaluated by pathologist to contain at least 20% tumor cells. Family history here is defined as the confirmed bowel cancer patient who has at least one family member (first and second-degree relatives) who had a history of tumor diagnosis. The immediate family member includes father, mother, brother(s), sister(s), son(s), daughter(s); second degree relatives include grandparent(s), uncle(s), aunt(s).

### Deoxy Ribonucleic Acid Isolation and Targeted Next-Generation Sequencing

The FFPE tissues and peripheral white blood cells were collected to extract DNA using QIAamp DNA FFPE Tissue Kit and DNeasy Blood and Tissue Kit (Qiagen, Inc.), respectively. And the purified gDNA was quantified using the Qubit 3.0 Fluorometer (Life Technologies, Inc.).

For the matched germline and tumor samples, 100 ng of DNA was shared with a Covaris E210 system (Covaris, Inc.) to obtain an average of 200 bp fragments. Accel-NGS 2S DNA Library Kit (Swift Biosciences, Inc.) and xGen Lockdown Probes kit (IDT, Inc.) were used for NGS library preparation of the tumor gDNA matched germline gDNA. The custom xGen Lockdown probe was synthesized by IDT, Inc. to target the exons and selected intronic regions of 499 genes (Gene list is provided in Supplementary Table S1).

### Interpretation of Pathogenicity of Germline Mutations and Calculation of Somatic Tumor Mutation Burden

Pathogenicity of germline mutations was defined and predicted based on the five-grade classification system according to the American College of Medical Genetics and Genomics (ACMG) Guidelines for the Interpretation of Sequence (Li et al., 2017). It was modified here that pathogenic/likely-pathogenic germline variants were depicted as P/LP and the variant of undetermined significance (VUS), benign, likely benign, and somatic mutations were defined as the non-pathogenic group (Non-P) in our results. Therefore, all mutations were categorized into P/LP or Non-P groups in this study.

Tumor mutation burden of each sample was calculated according to a published and the method of Chalmers et al (2017).

### Data Processing

Germline mutation data and incidence rates were obtained from the ChinaMAP database (http://www.GenomAD.org). The Cancer Genome Atlas (TCGA) database (https://tcga-data.nci.nih.gov/tcga/) provides several expression profiles and mutation data of CRC, as well as corresponding clinical data. Gene Ontology (GO) and the Kyoto Encyclopedia of Genes and Genomes (KEGG) pathway terms were considered statistically significant when FDR < 0.01. CIBERSORT was used for evaluating diverse immune cell types in the cancer microenvironment. The violin software package was used to visualize differentially infiltrated immune cells between the two groups through the Wilcoxon test.

### Statistical Analysis

Statistical analyses were performed using the Statistical Package for the Social Sciences (SPSS) statistical package and Graphpad (Prism 8). Student’s t-test was performed when two groups were compared, and ANOVA and post hoc tests were performed when three or more groups were compared. Gene prevalence between different groups was analyzed by Chi-Square test or Fisher exact test under/with a dominant model. A two-sided *p* value of less than 0.05 was considered to be statistically significant.

## Results

### Demographic Characteristics and Landscape of Mutational Profiles in Chinese Patients With Bowel Cancer

Briefly, paired tumor/germline analysis was conducted using a customized next generation sequencing (NGS) panel of 499 selected genes (Supplementary Table S1). Somatic variants were determined by comparing the data between tumor and blood samples and all participants were included as a result of successful germline sequencing, which resulted in an evaluable population of 573 patients with bowel cancer. The demographic, clinical, and pathological characteristics of this patient cohort are shown in Table 1. In this cohort (*n* = 573), 21.64% (*n* = 124) participants who were diagnosed with bowel cancers were < 50 years old and 78.36% (*n* = 449) > 50 years old. Approximately 39.62% (*n* = 227) patients were female, 66.32% (*n* = 380) exhibited colon cancer, 27.4% (*n* = 157) owned rectal cancer, and 52.53% (*n* = 301) patients were reported to have the stage IV tumors. A total of 108 (18.85%) participants had one or more first-degree relatives with a history of tumor diagnosis, and 13 (2.27%) had one or more second-degree relatives with a history of tumor diagnosis.

**TABLE 1:** Description of cohort.

Characteristic	Subgroups	Total evaluable cohort, no. (%)	
No. of participants	573		
Age, year	<50	124	21.64%
	≥50	449	78.36%
Sex	female	227	39.62%
	male	346	60.38%
Family history	Yes*	121	21.11%
	First-degree	108	18.85%
	Second-degree	13	2.27%
	No	275	48%
	NA	177	30.89%
Stage	Ⅰ	7	1.22%
	Ⅱ	202	35.25%
	Ⅲ	63	10.99%
	Ⅳ	301	52.53%
Tumor Location	Colon cancer	380	66.32%
	Rectal cancer	157	27.40%
	Duodenal Cancer	27	4.71%
	Small bowel cancer	6	1.05%
	Cecal cancer	3	0.52%

Yes*: the confirmed colorectal cancer patient has at least one family member (first- and second-degree relatives) who had a history of tumor diagnosis.

### Characteristics of Pathogenic Germline Mutations in the Chinese Cohort and Their Impact on Bowel Cancer Risk

Overall, 47 patients were found to carry P/LP germline mutations (Table 2). The age and sex of the patients were not associated with the presence or absence of a P/PL germline mutation (*p* = 0.19 and *p* = 0.21, respectively, Table 2). Interestingly, the ratio of patients with bowel cancer with at least one family member (first- and second-degree relatives) with family history of cancer(s), such as colon, breast, endometrium, ovary, and/or pancreas was significantly higher in the P/LP group than that of the Non-P group (*p* = 0.037, Table 2).

**TABLE 2:** The summary of clinicopathological and history information for bowel cancer patients with or without distinct germline mutation pathogenicity.

Characteristic	Subgroups	P/LP (N = 47)		Non-pathogenic (N = 526)		*p* Value
Age, year	<50	14	29.79%	110	20.91%	0.194
	≥50	33	70.21%	416	79.09%	—
Sex	female	23	48.93%	204	38.78%	0.213
	male	24	51.07%	322	61.22%	—
Family history	Yes*	14	29.79%	107	20.34%	0.037
	First-degree	12	25.53%	96	18.25%	—
	Second-degree	2	4.26%	11	2.09%	—
	No	15	31.91%	260	49.43%	—
	NA	18	38.30%	159	30.23%	—

Yes*: the confirmed colorectal cancer patient has at least one family member (first and second degree relatives) who had a history of tumor diagnosis.

**FIGURE 1:**
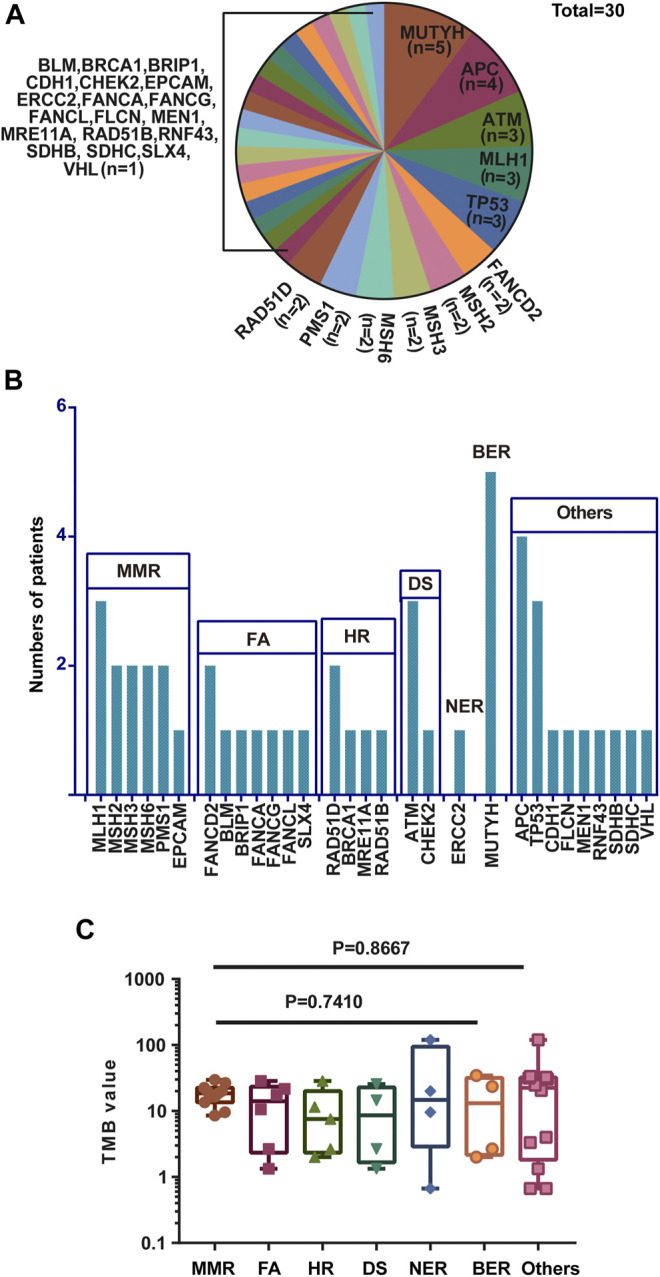
Gene names, functions, and number of variations of all P/LP germline mutations. **(A)** Gene names and the ratio of mutations of P/LP germline variations. **(B)** Specific gene mutation number with each DDR pathway in the P/LP group. **(C)** Comparison of the TMB with somatic mutations among different pathways in the P/LP group. pathogenic/likely-pathogenic: P/LP; DDR: DNA damage response; TMB, tumor mutation burden; HR, homologous recombination; FA, fanconi anemia; MMR, mismatch repair; NER, nucleotide excision repair; BER, base excision repair; DS, DNA sensor.

**FIGURE 2:**
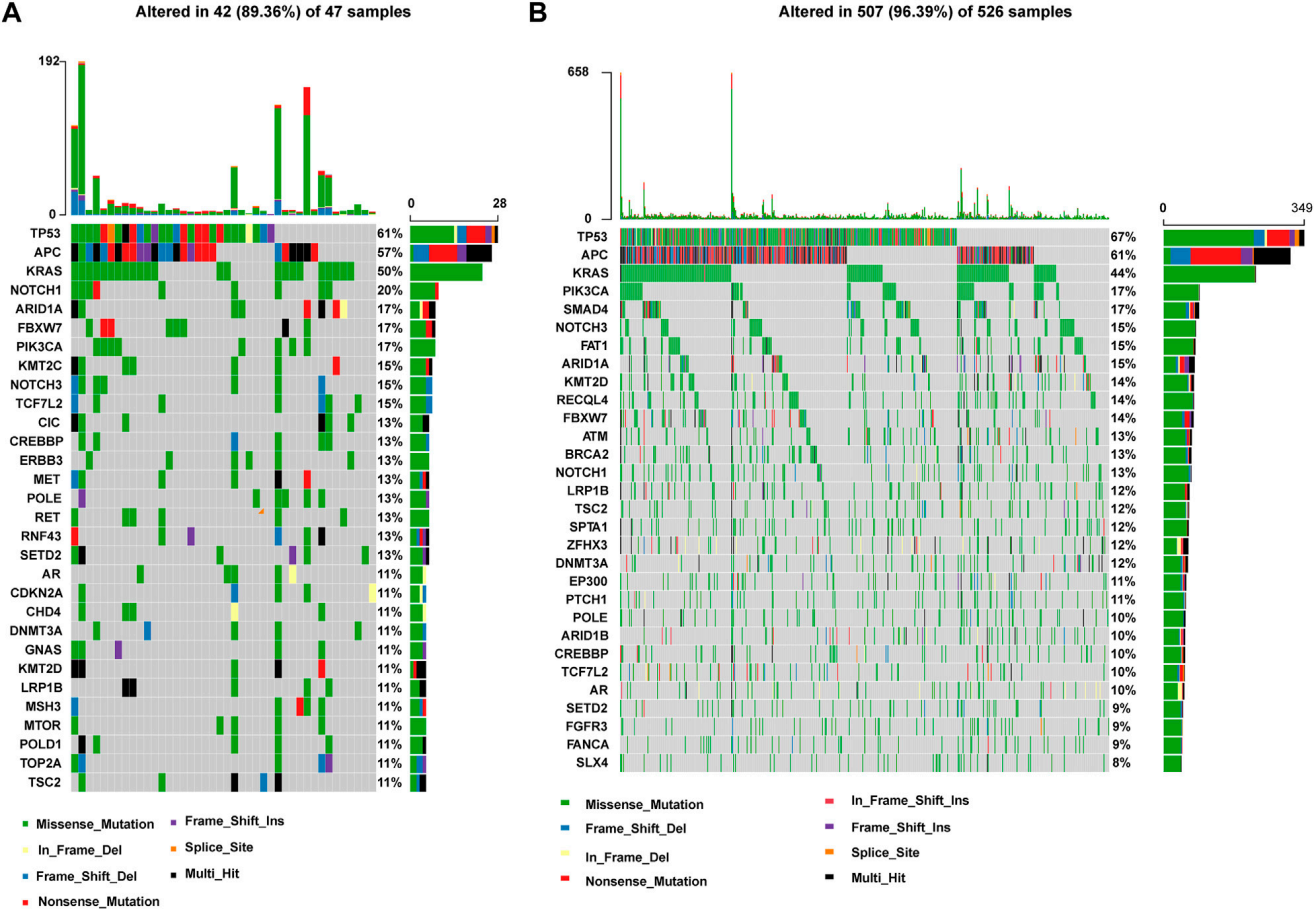
Heatmap of full SNV and INDEL somatic mutation for patients with P/LP **(A)** or Non-P **(B)** germline mutations. **(A)** Somatic mutation spectrum for 47 patients with P/LP germline mutations. **(B)** Somatic mutation spectrum for 526 patients with Non-P germline mutations. Details of mutations are labeled beneath each panel, and somatically mutated genes are listed in the left of the figures. The right bars represent the overall number of mutations for each gene. *Y*-axis above each panel represents the number of somatic mutations detected for each patient. Colors represent mutation types as indicated by the figure legend.

A total of 30 genes with P/PL germline variants were detected among 47 patients (Figure 1A). Besides, this study identified 5 out of 47 patients who carried *MUTYH* P/LP mutations (5/47), followed by *APC* (4/47) *MLH1* (3/47), *TP53*(3/47), and *ATM* (3/47) (Supplementary Table S2, Figure 2A). Moreover, it was found that 8 patients carried LS related mutations (3 *MLH1*, 2 *MSH2*, 2 *MSH6*, and 1 *EPCAM*) and 39 carried non-LS mutations (Figure 1A). These findings were consistent with those reported in the previous studies (Latham et al., 2019). Notably, certain novel P/LP mutations were present, including those in *FANCD2, RAD51D, BLM, CDH1, FLCN, MEN1, SDHB,* and *SLX4* were newly discovered in our cohort and have been rarely reported in the previous publications. The detailed distribution data and information on the germline mutations are presented in Supplementary Table S2. The functions of these genes with newly discovered P/LP mutations were mainly involved in DNA damage repair pathways (DDR-related genes were shown in Supplementary Table S3) and exerted a very broad impact. They included the following components: i) Those contributing to homologous recombination (HR), such as *BRCA1, RAD51D, MRE11A*, and *RAD51B*; ii) Those involved in Fanconi anemia (FA), such as *FANCG, FANCA, SLX4, BLM, FANCD2, FANCL,* and *BRIP1*; iii) Those involved in base excision repair (BER), such as *MUTYH*; iv) those involved in nucleotide excision repair (NER), such as *ERCC2*; v) those involved in mismatch repair (MMR), such as *MLH1, MSH2, MSH3, MSH6, PMS1,* and *EPCAM*; vi) those contributing to DNA sensor (DS), such as *ATM* and *CHEK2* (Figure 1B).

Mutations in the DNA damage repair genes increase the risk of subsequent mutations and therefore confer high cancer susceptibility. Previous studies verified the association between alterations in 34 DDR genes that exhibited higher TMB levels in urothelial cancer and demonstrated that DDR inactivation was associated with higher levels of TMB (Wang et al., 2018). Therefore, the TMB of CRC patients harboring germline mutations was compared among different DDR subgroups. Although there were no statistically significant differences with regard to the incidence of TMB in these pathways, the average value of MMR was the highest with the exception of the group of others, while HR exhibited the lowest (Figure 1C). This phenomenon is in line with the previous studies showing that over 90% of MMR-deficient tumors exhibit high TMB levels (Stadler et al., 2016). In addition, the data indicated that the majority of germline mutations were located in or affecting protein functional domains and that they may have a significant impact on protein function (Supplementary Figure S1).

To investigate the risk of bowel cancer in individuals carrying P/LP germline mutations, the mutation prevalence of all germline mutations was searched in the total population and in different populations derived from the China_MAPs database (Table 3). Eight genes were significantly associated with CRC compared with control subjects derived from the China_MAPs database. These included the following: i) *APC*, with mutations in 0.70% of cases and in 0.01% of control subjects (OR, 74.32); ii) *ATM*, with mutations in 0.52% of cases and in 0.03% of control subjects (OR, 18.56); iii) *MLH1*, with mutations in 0.52% of cases and in 0.01% of control subjects (OR, 111.50); iv) *FANCD2*, with mutations in 0.35% of cases and in 0.03% of control subjects (OR, 12.35); v) *MSH3*, with mutations in 0.35% of cases and in 0.04% of control subjects (OR, 9.27); vi) *MSH6*, with mutations in 0.35% of cases and in 0.01% of control subjects (OR, 74.07); vii) *PMS1*, with mutations in 0.35% of cases and in 0.30% of control subjects (OR, 10.59); viii) *RAD51D*, with mutations in 0.35% of cases and in 0.01% of control subjects (OR, 74.07) (Table 3). These findings suggested that these P/LP germline mutations were risk factors for the development of bowel cancer.

**TABLE 3:** Comparison of mutation carriers by panel gene between colorectal cancer cases and China_MAPs control cases.

	Cases			China_MAPs Cases			Cancer Risk	
Genes	Cases With Mutations, No	Individuals Tested, No	Carrier Frequency, %	Controls With Mutations, No	Individuals Tested, No	Carrier Frequency, %	Odds Ratio(95%CI)	*p* Value
Genes Significantly Associated with Colorectal Cancer								
*APC*	4	573	0.70	2	21,176	0.01	74.32 (10.63–833.35)	˂0.001
*ATM*	3	573	0.52	6	21,176	0.03	18.56 (3.00–87.19)	0.001
*MLH1*	3	573	0.52	1	21,176	0.01	111.50 (8.92–5,589.64)	˂0.001
*FANCD2*	2	573	0.35	6	21,176	0.03	12.35 (1.22–69.42)	0.02
*MSH3*	2	573	0.35	8	21,176	0.04	9.27 (0.96–46.56)	0.03
MSH6	2	573	0.354	1	21,176	0.01	74.07 (3.85–4,220.60)	0.002
*PMS1*	2	573	0.35	7	21,176	0.03	10.59 (1.07–55.73)	0.02
*RAD51D*	2	573	0.35	1	21,176	0.01	74.07 (3.85–4,220.60)	0.002
Genes Not Significantly Associated with Colorectal Cancer								
*BLM*	**1**	573	0.17	11	21,176	0.05	4.62(0.10–34.60)	3.36
*BRCA1*	**1**	573	0.17	8	21,176	0.04	5.29 (0.12–41.26)	4.62
*BRIP1*	1	573	0.17	7	21,176	0.03	12.33 (0.23–153.20)	5.29
*CHEK2*	1	573	0.17	3	21,176	0.01	18.50 (0.31–354.76)	12.33
*EPCAM*	1	573	0.17	2	21,176	0.01	6.17 (0.13–50.98)	18.50
*ERCC2*	1	573	0.17	6	21,176	0.03	5.29 (0.12–41.26)	6.17
*FANCA*	1	573	0.17	7	21,176	0.03	9.25 (0.19–93.72)	5.29
*FANCL*	1	573	0.17	4	21,176	0.02	36.98 (0.47–2,826.49)	9.25
*SLX4*	1	573	0.17	9	21,176	0.01	4.11 (0.09–29.76)	36.98
*VHL*	1	573	0.17	3	21,176	0.04	12.33 (0.23–153.20)	4.11
*MEN1*	1	573	0.17	1	21,176	0.01	4.62(0.10–34.60)	12.33

Furthermore, the bowel cancer germline mutation frequency found in the present study was compared with that reported in other studies including the investigations performed in Japan (Fujita et al., 2020) and in America (Yurgelun et al., 2017). For the incidence of *APC* and *MUTYH* in the Japanese cohort were significantly lower than those noted in the present (*p* = 0.02, *p* < 0.001 respectively). The P/PL prevalence did not differ significantly of genes including *MLH1, MSH2, MSH6, BRCA1, TP53, CHEK2, ATM,* and *BRIP1* among the three cohorts investigated. (Table 4).

**TABLE 4:** Comparison of germline mutation carriers with specific genes among different countries.

	China		Japan			America		
Genes	Individuals Tested, No	Carrier Frequency, %	Individuals Tested, No	Carrier Frequency, %	China vs. Japan *p* Value	Individuals Tested, No	Carrier Frequency, %	China vs. America *p* Value
*APC*	573	0.70	12,503	0.16	0.02	1,058	0.47	0.73
*MUTYH*	573	0.87	12,503	0.10	˂0.001	1,058	1.80	0.20
*MLH1*	573	0.52	12,503	0.28	0.23	1,058	1.18	0.20
*MSH2*	573	0.35	12,503	0.29	0.68	1,058	0.66	0.51
*MSH6*	573	0.35	12,503	0.31	0.70	1,058	0.57	0.72
*BRCA1*	573	0.17	12,503	0.17	1.00	1,058	0.28	1.00
*TP53*	573	0.52	12,503	0.15	0.07	1,058	0.09	0.13
*CHEK2*	573	0.17	12,503	0.12	0.51	1,058	0.19	1.00
*ATM*	573	0.52	12,503	0.37	0.47	1,058	0.95	0.56
*BRIP1*	573	0.17	12,503	0.14	0.57	1,058	0.28	1.00

### Molecular Analysis of Somatic Mutations of Patients With Bowel Cancer Carrying Germline P/LP Mutations

The relationship between germline mutation carriers and patients with somatic mutations has been studied in other cancer types, such as lung cancer (Liu et al., 2020). However, the connection of germline variations and somatic mutations in bowel cancer has not been explored in detail. The somatic mutation spectrum was classified by pathogenicity/likely pathogenicity of germline mutations for all patients with bowel cancer (P/LP and Non-P groups) (Figures 2A,B). *TP53, APC, KRAS, NOTCH1, ARID1A, FBXW7, PIK3CA, KMT2C, NOTCH3,* and *TCF7L2*, were found to be the top 10 mutated genes in the P/LP group. With regard to the Non-P group, the top 10 mutated genes were *TP53, APC, PIK3CA, SMAD4, NOTCH3, FAT1, ARID1A, KMT2D, RECQL4,* and *FBXW7.* According to the different mutation classification categories, the missense mutation was the one that obtained the highest proportion in the bowel cancer samples in the presence or absence of germline variants.

**FIGURE 3:**
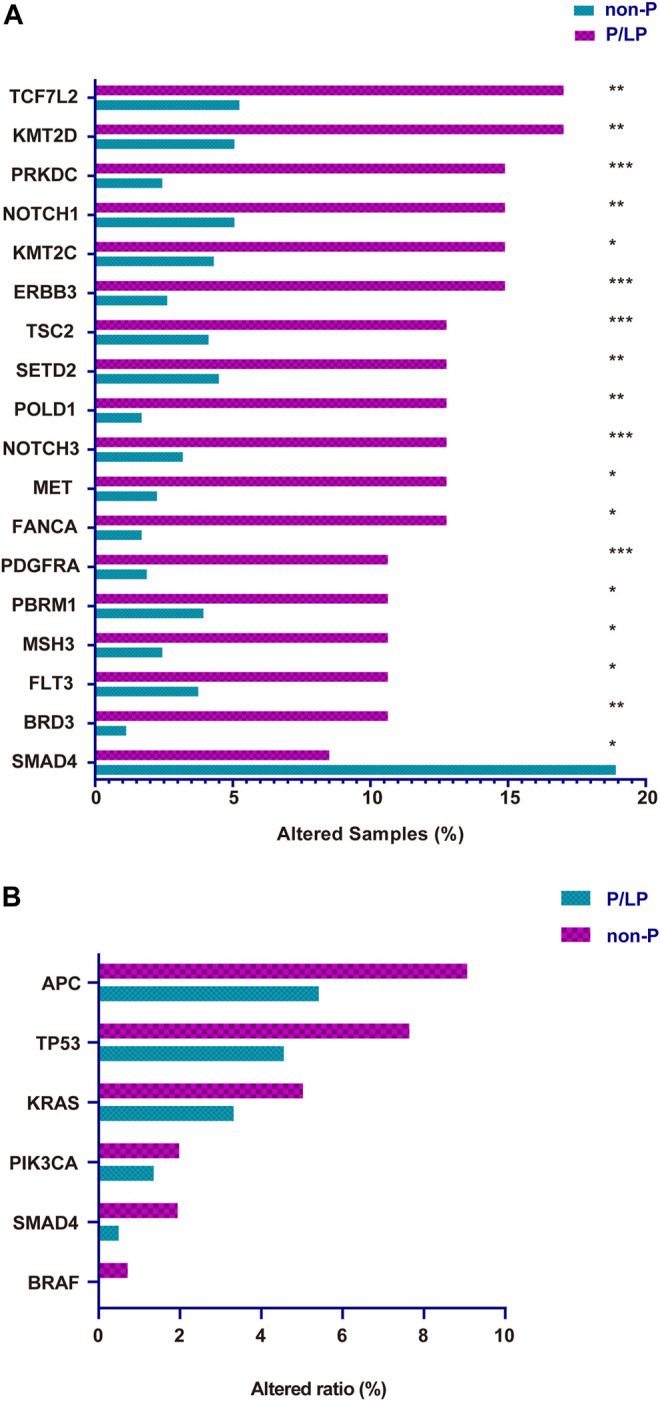
The somatic gene variation rate between P/LP and Non-P groups for all patients in this study. **(A)** Differential expressing Mutation genes in P/LP and Non-P groups. **(B)** Comparison of the variation rate (mutational frequency) for driver genes with somatic mutations between P/LP and Non-P groups. P/LP: pathogenic/likely-pathogenic; Non-P: non-pathogenic.

To determine the presence of germline variants in the patients examined, the comparison of somatic alterations was conducted between P/LP and Non-P groups, and the results revealed dramatic differences (*p* < 0.001) in several gene mutations between these two groups (Figure 3A). A total of 17 gene mutations such as *TCF7L2, KMT2D, PRKDC, NOTCH1, KMT2C, ERBB3,* and *TSC2*, and others were more common in patients with bowel cancer with P/LP germline mutations; strikingly, *SMAD4* exhibited the opposite prevalence and its mutation frequency in the Non-P group was significantly higher than that in the P/LP group (Figure 3A). Subsequently, Gene Ontology (GO) and Kyoto Encyclopedia of Genes and Genomes (KEGG) enrichment analyses were further conducted to explore the biological roles of the identified differential genes. GO as well as KEGG enrichment analysis revealed that these genes were mainly enriched in the biological process (BP) terms, such as PI3K signaling, DNA recombination, HR, and MMR (Supplementary Figure S2). Furthermore, the identification of specific driver genes in the present study has been previously reported (Eklöf et al., 2013; Dow et al., 2015). The mutation rate (frequency) of *APC* was the highest among all genes both in the P/LP and non-P groups followed by the mutation rate of *TP53*, *KRAS*, *PIK3CA*, *SMAD4* and *BRAF* (Figure 3B).

### Association Between Tumor Mutation Burden and the Incidence of DNA-Damage Repair Mutations in the Chinese Cohort

**FIGURE 4:**
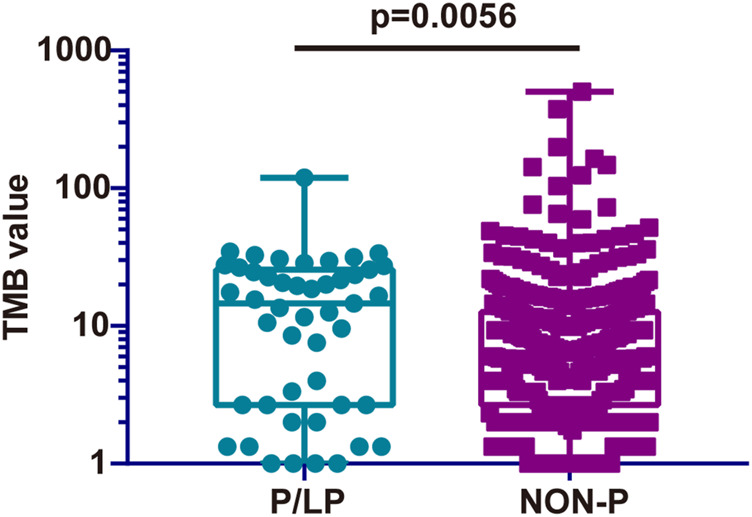
Comparison of the TMB from somatic mutations of the P/LP and the Non-P groups. P/LP: pathogenic/likely-pathogenic; Non-P: non-pathogenic; TMB, tumor mutation burden.

**FIGURE 5:**
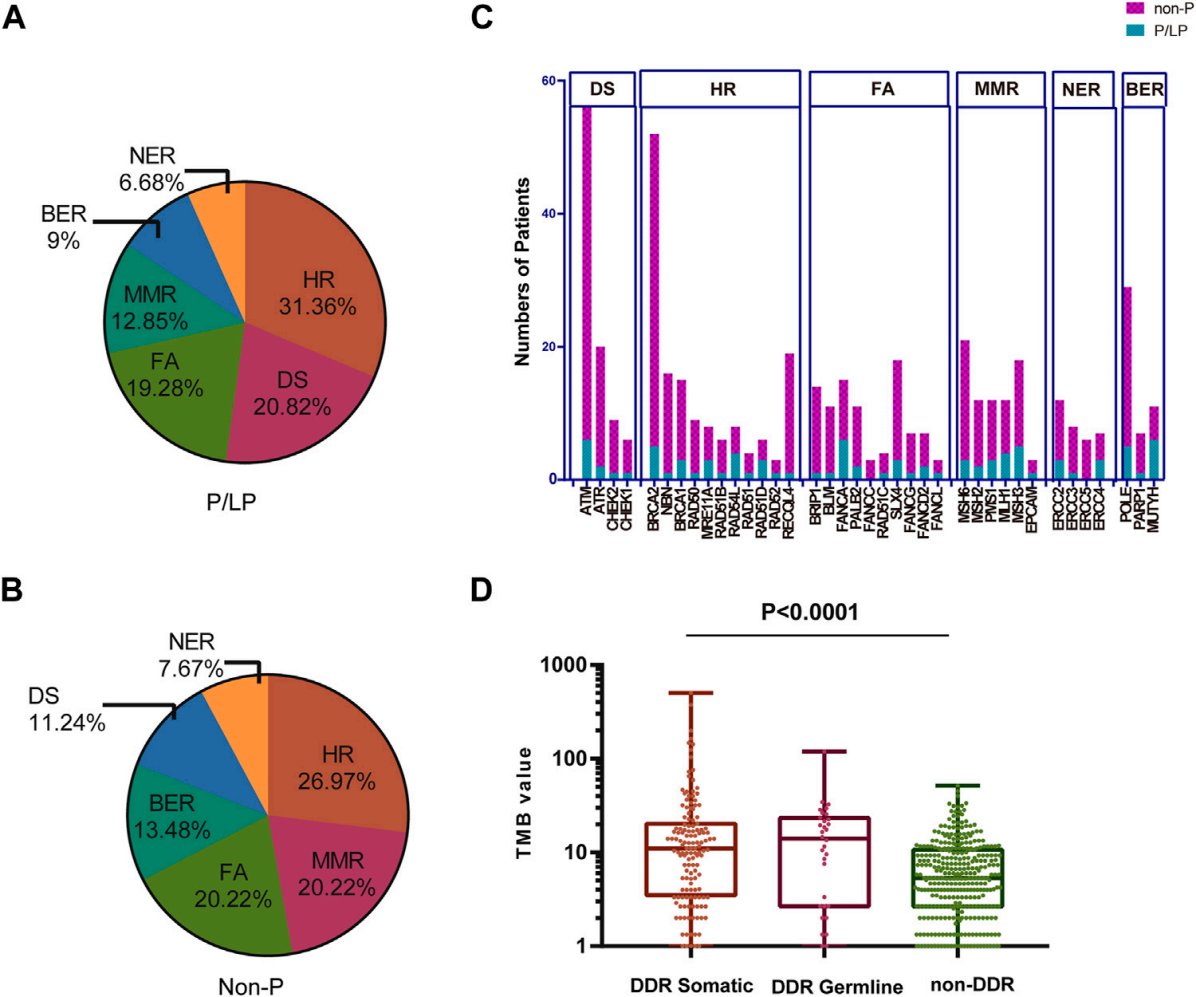
Percentage of patients with bowel cancer harboring DDR gene mutations and the TMB in our cohort. The proportion of different DDR pathways in P/LP **(A)** and Non-P groups **(B)**. **(C)** Comparison of the number of patients with gene mutations in DDR pathways for P/LP and Non-P groups. **(D)** Comparison of the TMB from somatic mutations of the DDR somatic, DDR germline, and non-DDR groups. P/LP: pathogenic/likely-pathogenic; DDR: DNA damage response; TMB, tumor mutation burden.

TMB is considered a vital biomarker in a variety of cancer types (Samstein et al., 2019; Wu et al., 2019), which may reflect the degree of genomic instability at the nucleotide level. Therefore, the TMB was initially compared in the P/LP and Non-P groups and the data indicated a significantly higher level of TMB in the P/LP group (14.56 vs. 6.39 mutations/Mb, *p* = 0.0056, Figure 4). Furthermore, the DDR system plays an important role in maintaining genome stability, based on this notion, a focused analysis was performed, specifically on the DDR-altered genes in all bowel cancer cases. In the P/LP group, DDR alterations were present in 68.1% (*n* = 32) of cases and were involved in the DDR pathways including HR (31.36%), DS (20.82%), FA (19.28%), MMR (12.85%), BER (9%) and NER (6.68%), respectively (Figure 5A). In the Non-P group, DDR gene mutations occurred in 30% (*n* = 157) of cases, including HR (26.97%), MMR (20.22%), FA (20.22%), BER (13.48%), DS (11.24%) and NER (7.67%) (Figure 5B). In the P/LP group, *FANCA, ATM,* and *MUTYH* were the most commonly altered DDR genes, followed by *BRCA2, MSH3,* and *POLE*; whereas in the Non-P group, *BRCA2* exhibited the highest DDR gene alterations, followed by *ATM, POLE,* and *MSH6* (Figure 5C). Furthermore, the highest average TMB value was observed in DDR-altered Non-P cases with bowel cancer compared with that of DDR-altered P/LP cases pf bowel cancer or Non-DDR altered cases (*p* < 0.001, Figure 5D). The data demonstrated that tumors with DDR mutations in the Non-P group exhibited increased genomic instability than that of the P/LP group. The lowest average TMB appeared in the non-DDR group, which is in line with the evidence reported in the previous studies.

### Comparison of Somatic Mutations Between the Chinese Cohort and the Independent The Cancer Genome Atlas Cohort

**FIGURE 6:**
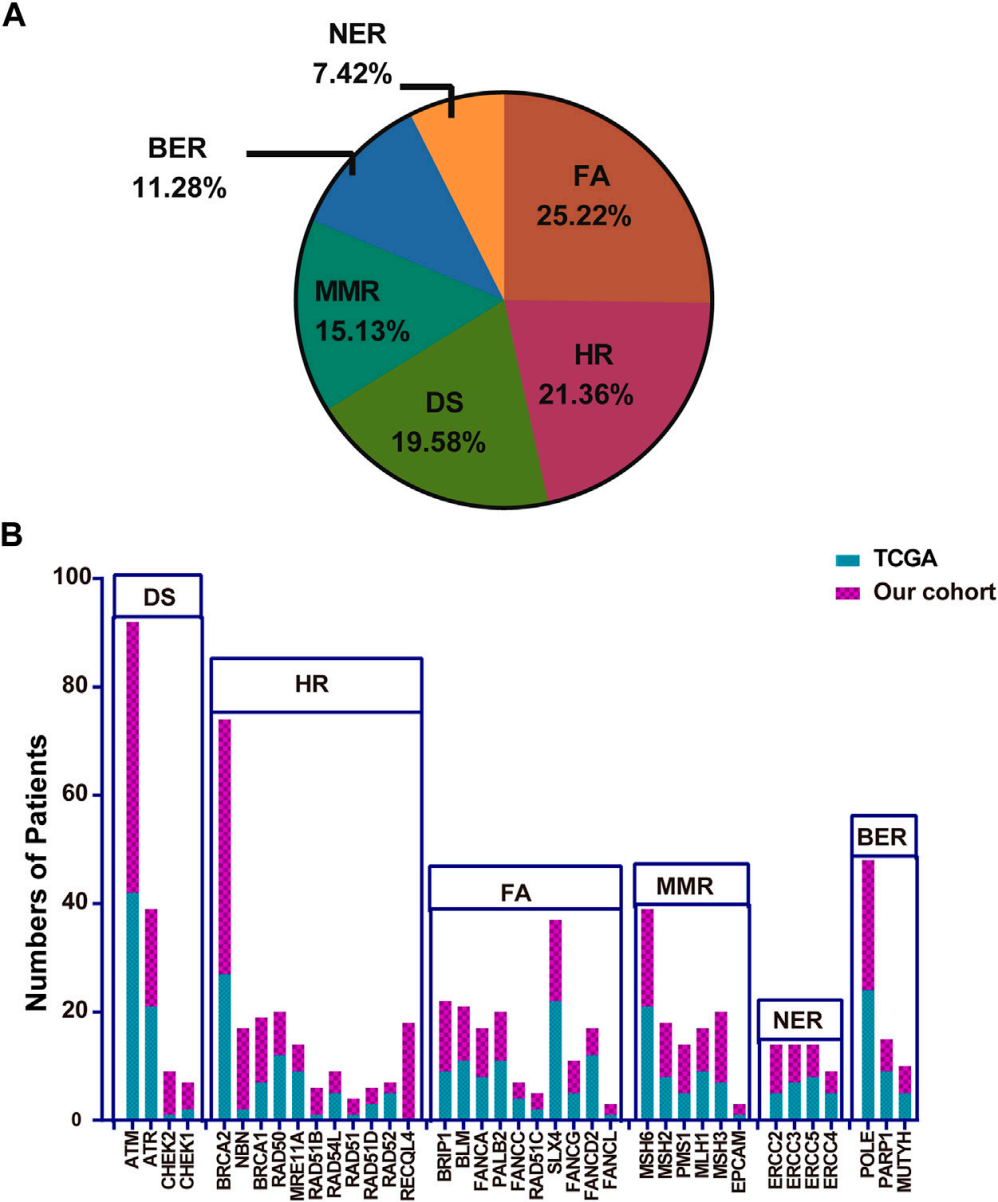
The proportion of the detailed DDR pathways **(A)** and the number of patients with specific DDR pathway genes in TCGA (B). TCGA: The Cancer Genome Atlas; DDR: DNA damage response.

A total of 223 bowel cancer samples with somatic mutation profiles were downloaded from the TCGA database. The relevant clinical information was listed in Supplementary Table S4. DDR gene mutation analysis was also conducted in the TCGA cohort, and the FA pathway accounted for the highest proportion (25.22%), while NER accounted for the lowest proportion (7.42%) (Figure 6A). Moreover, a similar finding was obtained regarding specific gene mutations in these pathways between TCGA cohort and the Chinese cohort, such as *ATM* (the highest mutation frequency) and *EPCAM* (relative lower mutation frequency) (Figure 6B).

**FIGURE 7:**
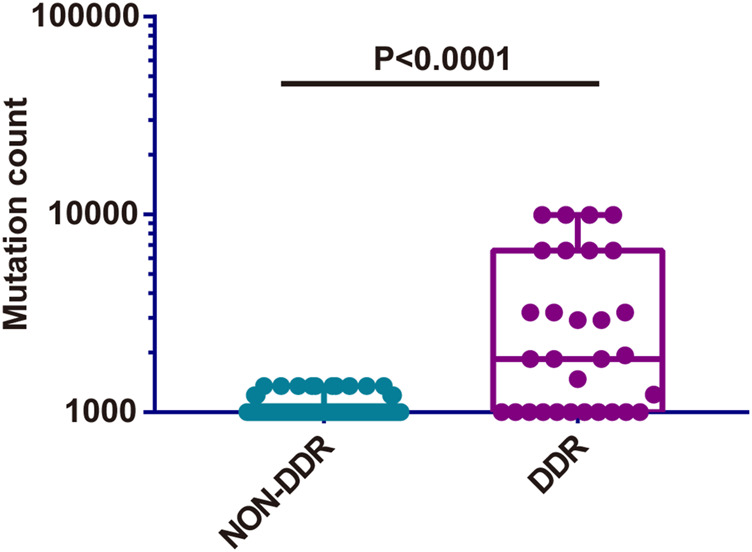
Comparison of somatic mutation count between non-DDR and DDR groups from TCGA cohort. DDR: DNA damage response; TCGA: The Cancer Genome Atlas.

Subsequently, the TMB in the Chinese cohort was compared with the mutation count in the TCGA cohort (TMB was not accessible and therefore the mutation count was adopted here). The selection of mutation genes with P/LP was performed *via* the cBioportal website. In concordance with the previous results, a significantly higher level of TMB was observed in bowel cancer harboring DDR somatic mutations compared with cases with non-DDR bowel cancer in TCGA cohort (*p* < 0.0001, Figure 7). A similar finding was obtained from the Chinese cohort (Figure 5D). Especially, although there was no statistically significant difference among the DDR pathways examined in the TCGA and the Chinese cohort, the lowest TMB or mutation count was observed in NER pathway. In addition, the HR pathway displayed an increased mutation count in the TCGA cohort, while the Chinese cohort harbored the highest TMB of somatic mutations in BER pathway (Supplementary Figure S3).

### Association Between DNA-Damage Repair Mutation and Immune Cell Infiltration Pattern in The Cancer Genome Atlas Cohort

**FIGURE 8:**
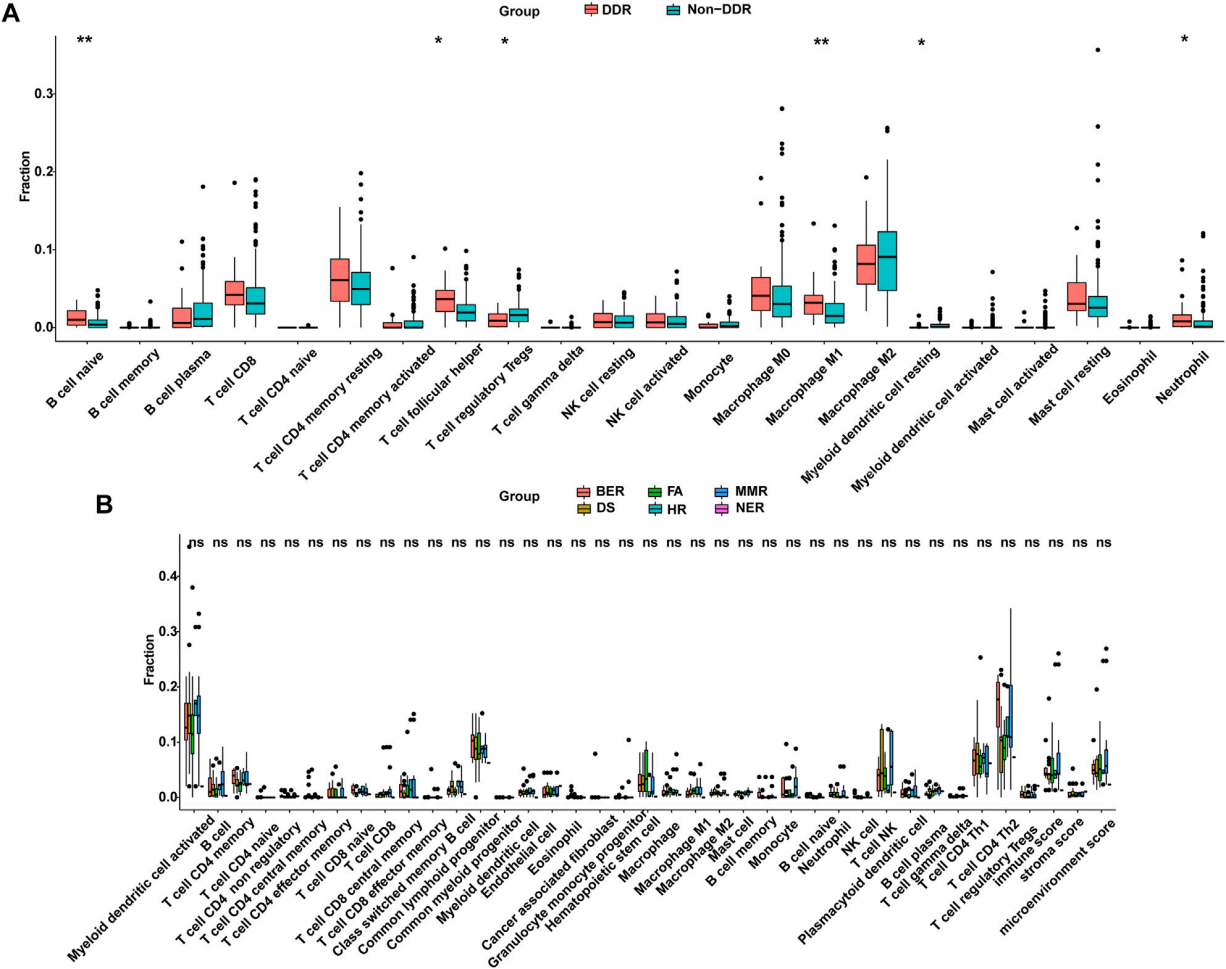
DDR was correlated with the infiltration of immune cells from the TCGA cohort. **(A)** Differentially infiltrated immune cells between DDR and non-DDR groups. **(B)** Comparison of infiltrated immune cells in different altered DDR pathways. DDR: DNA damage response; TCGA: The Cancer Genome Atlas.

Previous study has demonstrated that mutations can generate novel peptide sequences, which may affect the immune response (Chalmers et al., 2017). The TMB noted in the DDR mutation group was higher in the Chinese cohort and the TCGA cohort (Figure 5D, Figure 7). Thus, higher immune cell abundance was expected in the DDR somatic mutation. By applying the CIBERSORT algorithm, the differential variation of immune cell infiltration was estimated in the DDR and non-DDR groups of bowel cancer. The Wilcoxon rank-sum test indicated that the proportion of B cell naive (*p* = 0.017), T cell follicular helper (*p* = 0.0069), Macrophage M1 (*p* = 0.0038), and Neutrophils (*p* = 0.0163) were significantly elevated in the DDR group. By contrast, the infiltration levels of T cell regulatory Tregs (*p* = 0.0291) and Myeloid dendritic cell resting (*p* = 0.0163) were lower in the DDR group (Figure 8A). However, no association was observed between the different DDR pathway alterations and immune cell abundance (Figure 8B).

### Association Between DNA-Damage Repair Mutations and Survival Outcomes

**FIGURE 9:**
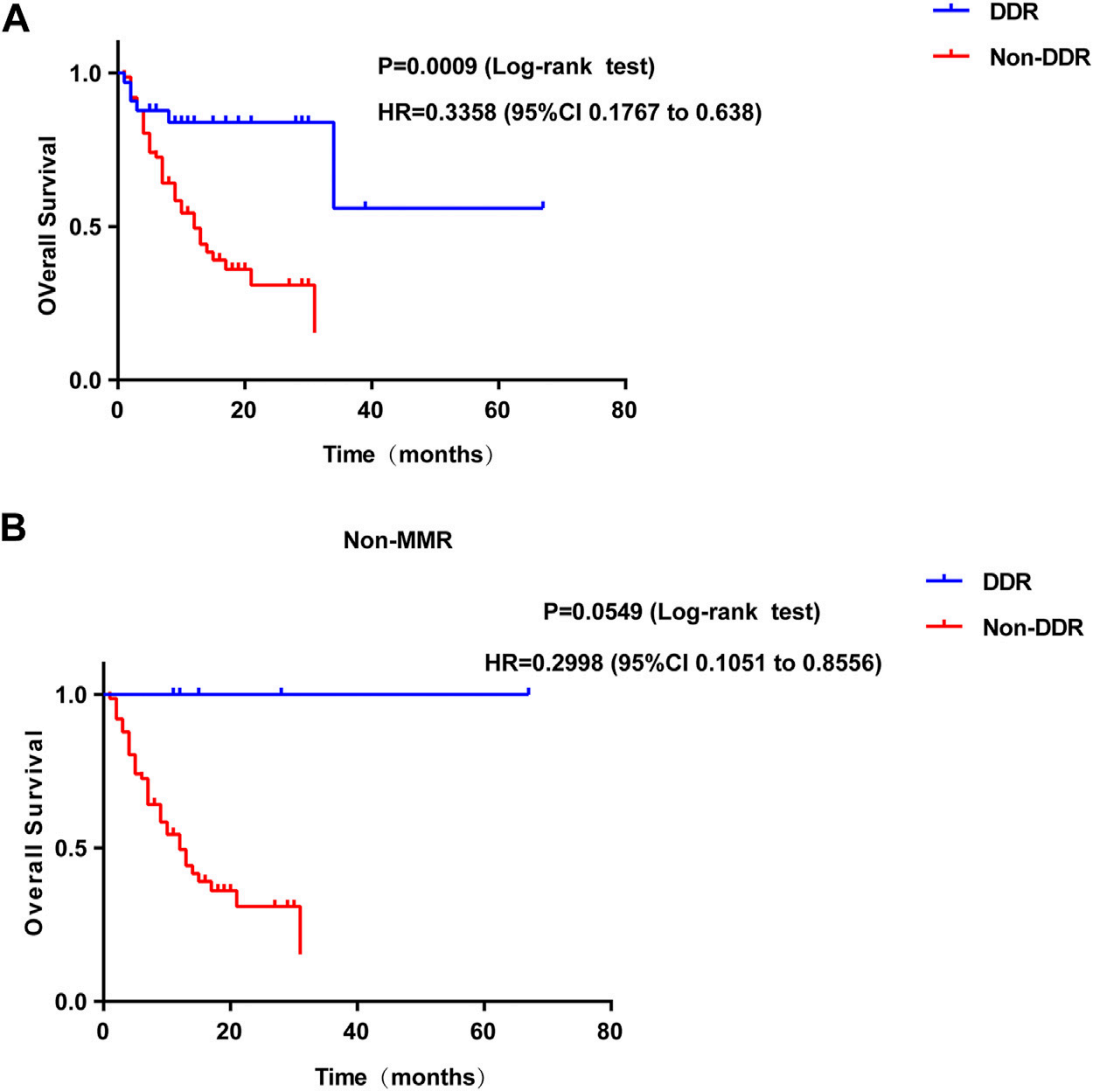
Survival curves of OS comparing DDR mutation **(A)** or DDR without MMR mutation **(B)** (blue) with Non-DDR (red) in colorectal patients from immunotherapy cohort. OS: overall survival; DDR: DNA damage response.

In addition, the present study further investigated whether the number of somatic mutations in the DDR genes of P/LP group was associated with improved survival following programmed cell death protein 1 (PD-1)/programmed death-ligand 1 (PD-L1) or cytotoxic T-lymphocyte-associated protein 4 immunotherapy in CRC patients. The clinical data of immunogenomic studies were downloaded from the cBioportal website. The overall survival (OS) was defined as the time from initial surgery to the date of death or last contact (censored). As expected, alteration in the mutation status of P/LP DDR conferred superior OS compared with patients without altered DDR gene(s) (HR, 0.3358; 95% CI, 0.1767 to 0.638; *p* = 0.0009) in the immunotherapy cohort (Figure 9A). However, patients with DDR mutations did not obtain a significantly prolonged OS when the MMR pathway was excluded (HR = 0.2998, 95%CI 0.1051 to 0.8556; *p* = 0.0549, Figure 9B).

## Discussion

Germline variants transmit genetic information that determines the heritability of complex disorders (McClellan et al., 2010). The presence of individually-rare but collectively common germline variants can explain a fraction of the missing genetic predisposition to bowel cancer. However, the major percentage of bowel cancer heritability is still not fully characterized, especially for the Chinese population. In the present study, an NGS-based analysis of germline mutations was performed for 573 Chinese patients with various stages of bowel cancer. The analysis provided a representative germline mutation landscape. The present study is the first to elucidate a more comprehensive germline mutation profile of Chinese patients with bowel cancer with the aim of identifying the novel candidate genes for hereditary bowel cancer. In addition, genetic testing and identification of germline mutations may have implications for the relatives of patients with bowel cancer because of the associated risks of CRC and other cancer types. In the present Chinese cohort, the ratio of patients with bowel cancer with at least one family member (first- and second-degree relatives) with tumor history was significantly higher in the P/LP group than that in the Non-P group, indicating that pathogenic cancer-predisposing variants were associated with the incidence of bowel cancer and resulted in familial clustering. On the other hand, the patients with bowel cancer and their family members with a history of other cancers were included in the family history examined in the current study indicating that the presence of pathogenic germline mutations increased the incidence of other cancer types.

Germline pathogenic variants in genes encoding for DNA mismatch repair proteins cause Lynch syndrome, which is considered the most prevalent form of hereditary bowel cancer (Arora et al., 2015). Classic hereditary bowel cancer syndromes, including Lynch syndrome are mainly due to germline mutations in *APC, MUTYH*, and the MMR genes (such as *MLH1, MSH2, MSH6,* and *PMS2*). The data reported in the current study demonstrated that the top three genes with the highest number of germline mutations were *MUTYH* (5/47), *APC* (4/47), and *MLH1* (3/47), which was consistent with the findings of previous publications (Esteban-Jurado et al., 2015; Reilly et al., 2019). Meanwhile, the recent study also revealed the most common mutated genes of *TP53, APC, KRAS, SMAD4, PIK3CA* etc., besides, the mutation frequencies of *TP53* and *APC* in the left CRC were significantly higher than that of right CRC (Huang et al., 2021). While the germline alterations in certain susceptibility genes were also detected in the bowel cancer samples including *FANCD2, CDH1, FLCN, MEN1, SDHB,* and *SLX4,* which have been rarely reported in the Chinese population. As is known, *FANCD2* is the frequently mutated gene in colorectal cancer (Offman et al., 2005). *CDH1* mutations are more predisposed to familial colorectal cancer (Richards et al., 1999). Besides, it was reported that the frameshift mutations in the *FLCN* exon 11 which would suppress the activation of *FLCN* could lead to the increased incidence of colorectal cancer (Nahorski et al., 2010). While *MEN1* was reported that it could be a novel driver causing the dysregulation of Wnt signaling pathway in colorectal cancer (Fennell et al., 2020). SDHB is the catalytic core of succinate dehydrogenase (SDH), of which dysfunction would exert an influence on the TGF-beta signaling pathway contributing to the colorectal cancer formation (Wang et al., 2016). Moreover, the mutation of the tumor suppressor gene SLX4 was recently shown to be associated with the early-onset of CRC in the population of Kazakhstan (Zhunussova et al., 2019).Among these germline variants, mutations in *SLX4, FANCD2,* and *FLCN* are associated with FA pathway alteration (provided by RefSeq, NCBI). In other geographical regions, germline mutations of CRC patients mainly occur in the HR, DS, NER, BER, and MMR pathways (Berginc et al., 2009; Yurgelun et al., 2017; Ma et al., 2018; Schneider et al., 2018; Fujita et al., 2020). Although the present study detected germline mutations in these DDR pathways, a higher number of genes were identified which were involved in the FA pathway compared with the previous studies, such as *FANCD2, FANCA, FANCG, FANCL,* and *SLX4*. The identification of a wider causative mutation in bowel cancer has implications that can apply to genetic counseling practices that are of vital importance for the family under investigation (Wells and Wise, 2017). Once established in a particular family with carriers and non-carriers, prevention strategies can be directed more precisely to those subjects carrying the causative mutation and who are therefore at risk of developing bowel cancer and other related malignancies.

The frequency of mutations queried in the China_MAPs database represents the frequency of a certain mutation site in the general population. The OR of the cases investigated in the present study suggested that the germline mutations were risk factors for bowel cancer. In this case-control study, mutations in 8 genes (*APC, ATM, MLH1, FANCD2, MSH3, MSH6, PMS1,* and *RAD51D*) were found to be associated with bowel cancer and were present in 3.5% of patients with bowel cancer. Mutations in *APC, ATM*, and *MLH1* were associated with the highest risks of bowel cancer. The frequency of latter two germline variants was also the highest among patients with bowel cancer (0.7, 0.52, and 0.52%, respectively). In addition to commonly mutated genes such as *APC, ATM,* and LS-related genes that have been previously reported (Bernstein and Concannon, 2017; Snowsill et al., 2017; Aghabozorgi et al., 2019), the current analysis further revealed significantly higher rates of *FANCD2* and *RAD51D* mutations in bowel cancer germline mutation carriers than those of the general population, suggesting that *FANCD2* and *RAD51D* can be considered as bowel cancer-susceptibility genes. FANCD2 is monoubiquinated in response to DNA damage, resulting in its localization to nuclear foci with other proteins (BRCA1 and BRCA2) involved in homology-directed DNA repair. Furthermore, *RAD51D* is involved in the homologous recombination and repair of DNA (provided by RefSeq, NCBI). However, these genes are rarely reported in bowel cancer. Therefore, the current results provide preliminary evidence of potential susceptibility genes that can be used for hereditary bowel cancer.

The integration of germline and somatic genomic data can provide insight into the mechanisms that drive tumor progression (Ramroop et al., 2019). Therefore, an in-depth integrated analysis was performed on germline and somatic NGS data derived from the patients with bowel cancer. The present study identified several distinct somatic mutation rates between the carriers of germline mutations and the non-carriers. For example, differential expression gene analysis indicated that the mutation incidence of specific genes, such as *TCF7L2, KMT2D, PRKDC,* and *NOTCH1* was significantly higher in the P/LP group than that of the Non-P group, suggesting a higher risk compared with subjects without germline mutations. It is interesting to note that the *SMAD4* mutation rate in the P/LP group was dramatically lower than that of the Non-P group. Mutations or deletions in this gene have been shown to result in the development of juvenile polyposis syndrome, whereas weak expression of *SMAD4* is known to associate with poor survival in patients with bowel cancer (Yan et al., 2016). The results in this study confirmed that *SMAD4* mutation mainly occurred in somatic mutation patients rather than germline carriers. One possible explanation for this outcome could be that the environmental factors mainly affected the Non-P germline mutation carriers.

It is noteworthy that somatic mutations in patients with P/LP germline mutations showed distinct characteristics in the present study. TMB, which is considered to be a predictive biomarker of immune checkpoint inhibitor (ICIs) (Wang and Li, 2019), may reflect the degree of genomic instability at the nucleotide level. The TMB in the P/LP group was significantly lower than that in the non-germline mutant group. It was speculated that somatic mutations in patients with P/LP germline mutations may be more focused on certain key genes and key pathways, whereas somatic mutations in patients without P/LP germline mutations may be more sporadic. Therefore, patients with P/LP germline mutations may be more likely to have abnormalities in key genes and pathways, leading to an increased risk of bowel cancer. Moreover, TMB in the non-altered DDR group was significantly lower than that noted in the altered DDR groups. In addition, higher TMB was observed in the DDR of the non-germline mutation group compared with that of the DDR germline mutation group. A possible interpretation is that the further alteration in DDR may induce a hypermutated phenotype with a higher TMB, which could be established as a biomarker of ICI treatment (Rizvi et al., 2015).

In the current comprehensive analysis, we also explored the association between DDR mutation and the number of immune cells among patients with bowel cancer from the TCGA cohort. The results found that the number of B cell naive, T cell follicular helper, Macrophage M1, and Neutrophil were elevated in the DDR group. Previous studies have demonstrated that the infiltration of B cells plays an important role in tumor immunotherapy (Cabrita et al., 2020; Helmink et al., 2020; Petitprez et al., 2020). Wei, et al. (2016) reported that B cell naive could suppress the antitumor adaptive immune response in the patients with ovarian cancer. An additional study proposed that B cell naive could be considered as a readily available and effective source of antigen-presenting cells in clinical research on tumor immunotherapy (Ren et al., 2014). Based on these findings, it was speculated that DDR alteration in bowel cancer might modulate the response to ICIs to a certain extent (Ren et al., 2014). In addition, the present study indicated that patients with DDR gene alterations were more likely to experience improved OS than patients with unaltered DDR genes. Although MMR mutations were excluded from the DDR pathway, patients with DDR alterations did not show a significantly prolonged OS, and the *p* value(*p* = 0.0549) is nearly close to the significance cutoff. The limited samples (*n* = 6) may contribute to this phenomenon. Therefore, it is suggested that the predicted value of this association is investigated further in larger data sets from randomized studies that have led to the FDA approval of several anti-PD-1/PD-L1 agents. DDR alterations may represent a useful predictive bowel cancer biomarker for of the patient response to anti-PD-1/PD-L1 provided these findings are validated in a larger cohort (Samstein and Riaz, 2018; Zhang et al., 2020).

The ratio of DDR pathway alteration and other molecular results varied between the TCGA cohort and the Chinese cohort. The baseline characteristics, such as sex and tumor stage, may also contribute to the distinction.

One limitation of the present study is that the germline mutations were unavailable from TCGA cohort. This cannot comprehensive assessment of the differences between P/LP germline mutation carriers and Non-P germline mutation carriers in distinct bowel cancer populations. In addition, although the mutations were identified in the genomic sequences, their exact effects on the altered protein function were not assessed. Despite these limitations, the results can still provide a reasonable basis for exploring the applications of the DDR germline mutations in the prognosis of hereditary bowel cancer.

In conclusion, the present study identified unique genomic and molecular characteristics such as TMB and DDR between P/LP germline alteration carriers and Non-P bowel cancer patients. A preliminary basis was provided for the assessment of a wider range of susceptibility genes in Chinese CRC patients. Moreover, the TCGA database indicated that a deeper understanding of the interactions between DDR and immune cell infiltration would be useful to further investigate the role of DDR in bowel cancer.

## Data Availability

The raw data of next generation sequencing in this study are deposited in: https://bigd.big.ac.cn/gsa-human/browse/HRA001620 (the Chinese National Genomics Data Center) repository, accession number: HRA001620. The control data from the China Maps database were downloaded from the following: http://www.GenomAD.org.
