# Supplementary figures and images for: Prevalence and Spectrum of Predisposition Genes With Germline Mutations Among Chinese Patients With Bowel Cancer
Source: Front Genet. 2022 Jan 27;12:755629. doi: 10.3389/fgene.2021.755629 (PMC8829568; doi:10.3389/fgene.2021.755629)

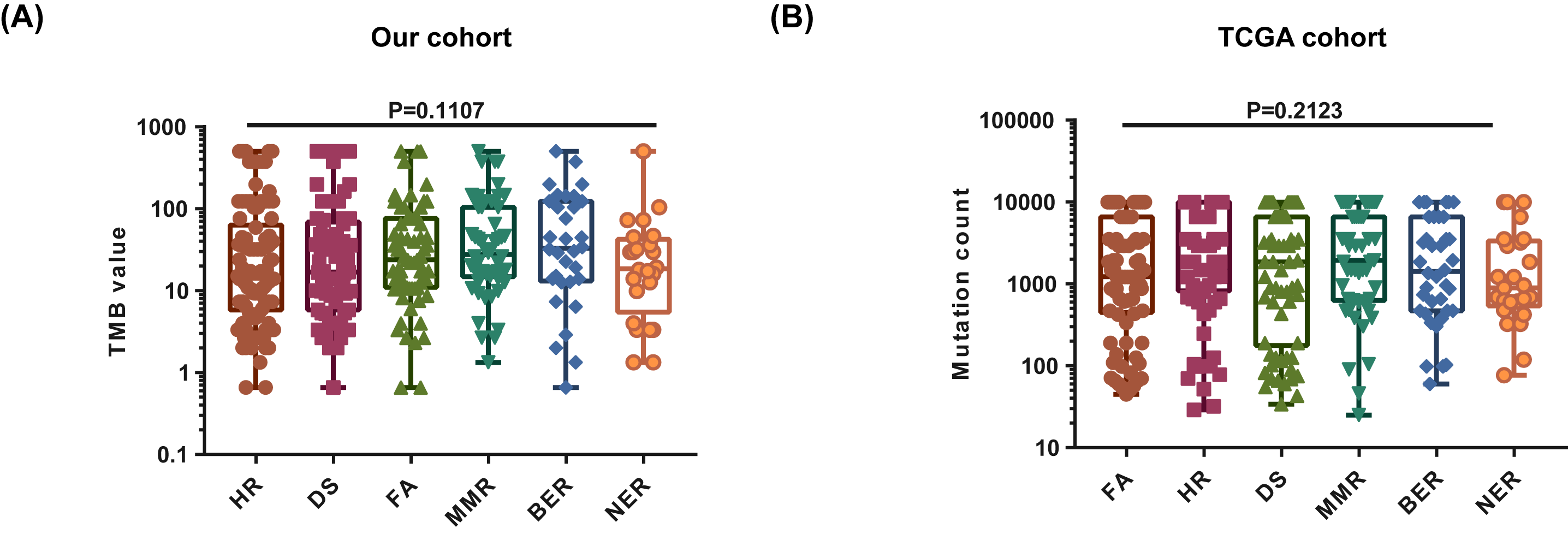

Supplement: Supplementary file 2 [file Image3.TIF]

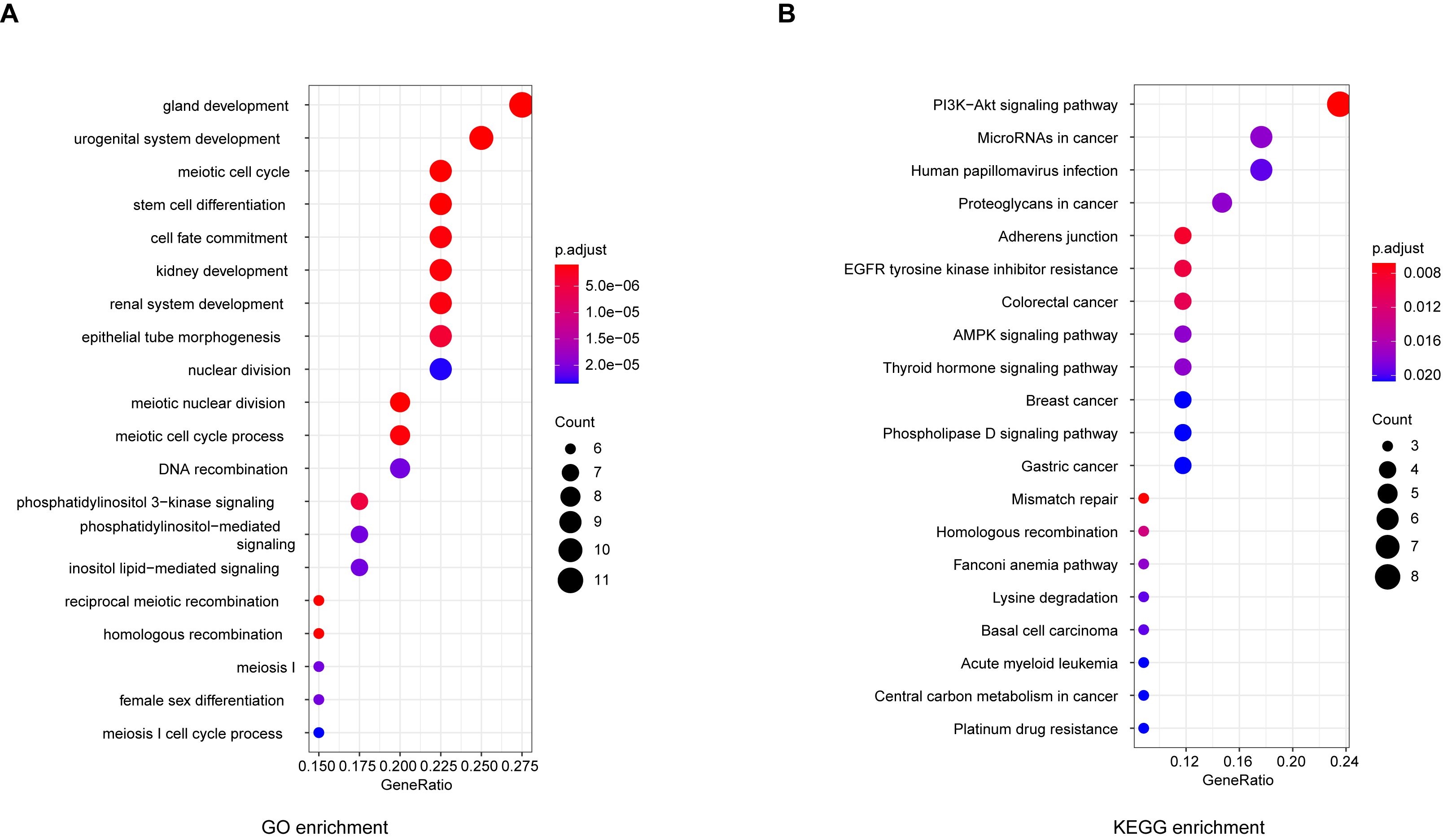

Supplement: Supplementary file 3 [file Image2.TIF]

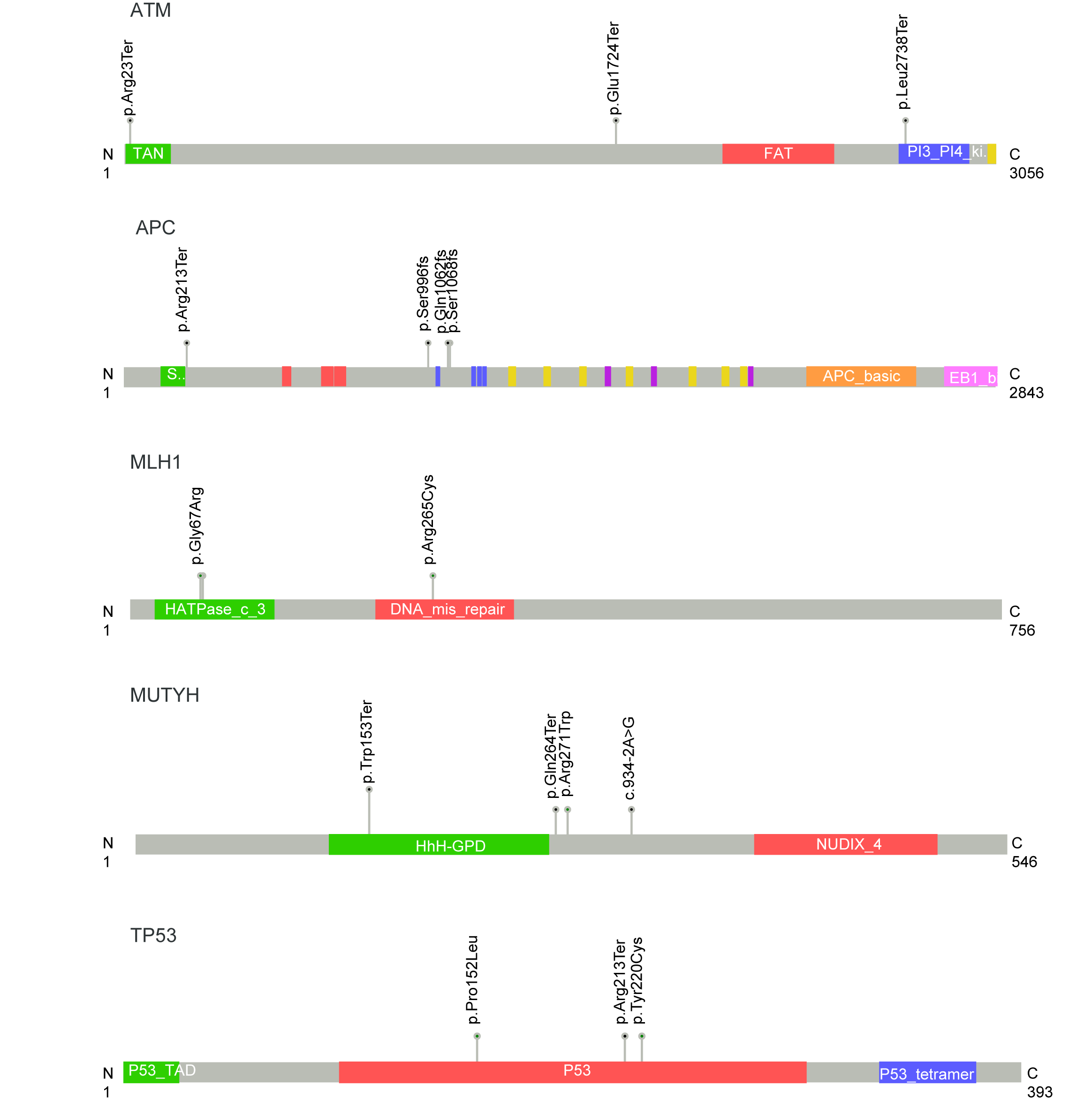

Supplement: Supplementary file 4 [file Image1.TIF]
