# Supplementary material for: Prevalence and Spectrum of Predisposition Genes With Germline Mutations Among Chinese Patients With Bowel Cancer
Source: Front Genet. 2022 Jan 27;12:755629. doi: 10.3389/fgene.2021.755629 (PMC8829568; doi:10.3389/fgene.2021.755629)
Supplement: Supplementary file 5 [file Table2.DOCX]

**Table S2. Detailed distribution data and information of germline mutations**

| **Sample** | **Sample type** | **Stage** | **gene** | **protein_**  **change** | **variant_allele_**  **frequency** | **mutation_type** | **clinical_significance** |
| --- | --- | --- | --- | --- | --- | --- | --- |
| 1 | Tissue | Ⅳ | *ACVR1B* |  | 0.387784 | splice | pathogenic |
| 2 | Blood | Ⅳ | *ABL1* | p.Arg89Trp | 0.006105 | nonsynonymous | pathogenic |
| 3 | Tissue | Ⅱ | *ABL1* | p.Arg460His | 0.137868 | nonsynonymous | pathogenic |
| 4 | Tissue | Ⅱ | *ABL1* | p.Gly97Trp | 0.117791 | nonsynonymous | pathogenic |
| 5 | Tissue | Ⅳ | *APC* | p.Glu1306Ter | 0.419948 | stopgain | pathogenic |
| 6 | Tissue | Ⅳ | *FOXP2* | c.673-1G>C | 0.015873 | splice | pathogenic |
| 7 | Tissue | Ⅱ | *ATR* | p.Cys2326Tyr | 0.119048 | nonsynonymous | likely pathogenic |
| 8 | Tissue | Ⅲ | *ATM* | p.Tyr1938Asp | 0.046142 | nonsynonymous | pathogenic |
| 9 | Tissue | Ⅳ | *APC* | p.Arg876Ter | 0.323413 | stopgain | likely pathogenic |
| 10 | Blood | Ⅳ | *APC* | p.Arg876Ter | 0.039636 | stopgain | likely pathogenic |
| 11 | Tissue | Ⅲ | *KRAS* | p.Gly12Val | 0.23435 | nonsynonymous | pathogenic |
| 12 | Tissue | Ⅲ | *ARID1A* | p.Gly655Ter | 0.1125 | stopgain | pathogenic |
| 13 | Tissue | Ⅱ | *EPAS1* | p.Ser474Thr | 0.010705 | nonsynonymous | pathogenic |
| 14 | Blood | Ⅳ | *APC* | p.Lys1308Ter | 0.706683 | stopgain | likely pathogenic |
| 15 | Tissue | Ⅲ | *ALOX12B* | p.Glu638Gly | 0.032787 | nonsynonymous | likely pathogenic |
| 16 | Tissue | Ⅱ | *ACVR1* | p.Ala488Ser | 0.08038 | nonsynonymous | likely pathogenic |
| 17 | Blood | Ⅳ | *APC* | p.Glu1554fs | 0.20263 | frameshift-insertion | pathogenic |
| 18 | Tissue | Ⅱ | *ARID1B* | p.Thr1218Ser | 0.040375 | nonsynonymous | likely pathogenic |
| 19 | Tissue | Ⅲ | *ARID1A* | p.Gln766Ter | 0.025172 | stopgain | pathogenic |
| 20 | Tissue | Ⅱ | *AXIN2* | p.Lys642fs | 0.121374 | frameshift-deletion | likely pathogenic |
| 21 | Tissue | Ⅳ | *BLM* | p.Asp1188fs | 0.478431 | frameshift-deletion | likely pathogenic |
| 22 | Tissue | Ⅳ | *ABL2* | p.Asn110Tyr | 0.285345 | nonsynonymous | likely pathogenic |
| 23 | Tissue | Ⅲ | *KRAS* | p.Gly12Asp | 0.075 | nonsynonymous | likely pathogenic |
| 24 | Tissue | Ⅱ | *APC* | p.Ser583Ter | 0.043532 | stopgain | likely pathogenic |
| 25 | Tissue | Ⅱ | *ARID1A* | p.Ser2156Arg | 0.105353 | nonsynonymous | pathogenic |
| 26 | Tissue | Ⅱ | *APC* | p.Ser1400Ter | 0.537884 | stopgain | pathogenic |
| 27 | Tissue | Ⅱ | *TP53* | p.Ser127fs | 0.343593 | frameshift-insertion | pathogenic |
| 28 | Tissue | Ⅳ | *APC* | p.Lys670fs | 0.183499 | frameshift-deletion | pathogenic |
| 29 | Tissue | Ⅰ、0 | *EGFR* | amplification | 19.67415364 | amplification | pathogenic |
| 30 | Blood | Ⅰ、0 | *ATRX* | p.Lys1936Arg | 0.003902 | nonsynonymous | pathogenic |
| 31 | Tissue | Ⅳ | *AR* | p.Glu128Lys | 0.034675 | nonsynonymous | pathogenic |
| 32 | Tissue | Ⅳ | *APC* | p.Ser1545Ter | 0.248 | stopgain | likely pathogenic |
| 33 | Tissue | Ⅳ | *APC* | p.Glu988Ter | 0.402505 | stopgain | pathogenic |
| 34 | Tissue | Ⅳ | *APC* | p.Ser1344Ter | 0.356337 | stopgain | pathogenic |
| 35 | Blood | Ⅳ | *SYK* | p.Lys387Arg | 0.007079 | nonsynonymous | likely pathogenic |
| 36 | Tissue | Ⅳ | *CHD3* | p.Pro598Arg | 0.079099 | nonsynonymous | pathogenic |
| 37 | Tissue | Ⅱ | *ATM* | p.Arg2244Thr | 0.068152 | nonsynonymous | pathogenic |
| 38 | Tissue | Ⅱ | *ERCC3* | p.Glu264del | 0.033585 | non-frameshift-deletion | pathogenic |
| 39 | Tissue | Ⅱ | *APC* | p.Thr1556fs | 0.19375 | frameshift-insertion | likely pathogenic |
| 40 | Tissue | Ⅳ | *BRD3* | p.Lys494Glu | 0.056289 | nonsynonymous | likely pathogenic |
| 41 | Tissue | Ⅲ | *APC* | p.Glu1397fs | 0.094241 | frameshift-deletion | likely pathogenic |
| 42 | Tissue | Ⅳ | *APC* | p.Arg876Ter | 0.117433 | stopgain | likely pathogenic |
| 43 | Tissue | Ⅲ | *APC* | p.Ser1344Ter | 0.17148 | stopgain | pathogenic |
| 44 | Tissue | Ⅰ | *APC* | p.Asp1498fs | 0.13347 | frameshift-deletion | pathogenic |
| 45 | Tissue | Ⅱ | *APC* | p.Leu1488fs | 0.34972 | frameshift-deletion | likely pathogenic |
| 46 | Tissue | Ⅱ | *APC* | p.Arg1450Ter | 0.151328 | stopgain | likely pathogenic |
| 47 | Tissue | Ⅳ | *APC* | p.Thr621fs | 0.091496 | frameshift-insertion | likely pathogenic |
