# Supplementary material for: Prevalence and Spectrum of Predisposition Genes With Germline Mutations Among Chinese Patients With Bowel Cancer
Source: Front Genet. 2022 Jan 27;12:755629. doi: 10.3389/fgene.2021.755629 (PMC8829568; doi:10.3389/fgene.2021.755629)
Supplement: Supplementary file 6 [file Table3.DOCX]

**Table S3. Gene list for the DDR gene panel used for mutation detection in this study**

| DDR Pathway | Genes | | | |
| --- | --- | --- | --- | --- |
| DS | *ATM* | *ATR* | *CHEK2* | *CHEK1* |
| HR | *BRCA2* | *NBN* | *BRCA1* | *RAD50* |
|  | *MRE11A* | *RAD51B* | *RAD54L* | *RAD51* |
|  | *RAD51D* | *RAD52* | *RECQL4* |  |
| FA | *BRIP1* | *BLM* | *FANCA* | *PALB2* |
|  | *FANCC* | *RAD51C* | *SLX4* | *FANCG* |
|  | *FANCD2* | *FANCL* |  |  |
| MMR | *MSH6* | *MSH2* | *PMS1* | *MLH1* |
|  | *MSH3* | *EPCAM* |  |  |
| NER | *ERCC2* | *ERCC3* | *ERCC5* | *ERCC4* |
| BER | *POLE* | *PARP1* | *MUTYH* |  |
