# Supplementary material for: Prevalence and Spectrum of Predisposition Genes With Germline Mutations Among Chinese Patients With Bowel Cancer
Source: Front Genet. 2022 Jan 27;12:755629. doi: 10.3389/fgene.2021.755629 (PMC8829568; doi:10.3389/fgene.2021.755629)
Supplement: Supplementary file 7 [file Table4.DOCX]

**Table S4. The summary of clinicopathological information in our cohort and TCGA**

| Characteristic | Subgroups | TCGA | | Our cohort | |
| --- | --- | --- | --- | --- | --- |
|  |  | (N=223) | | (N=573) | |
| Age, year | <50 | 19 | 8.52% | 65 | 12.36% |
|  | ≥50 | 204 | 91.48% | 323 | 61.41% |
|  | NA | - | - | 138 | 26.23% |
| Sex | female | 107 | 47.98% | 164 | 31.18% |
|  | male | 116 | 52.02% | 255 | 48.48% |
|  | NA | - | - | 107 | 20.34% |
| Stage | Ⅰ | 9 | 4.04% | 7 | 1.22% |
|  | Ⅱ | 18 | 8.07% | 202 | 35.25% |
|  | Ⅲ | 47 | 21.08% | 63 | 10.99% |
|  | Ⅳ | 149 | 66.82% | 301 | 52.53% |
